# Integrative medicine in treating post-stroke depression: Study protocol for a multicenter, prospective, randomized, controlled trial

**DOI:** 10.3389/fpsyg.2022.923506

**Published:** 2022-08-29

**Authors:** Jing Chen, Ke Shen, Lijuan Fan, Hantong Hu, Tieniu Li, Yiting Zhang, Hong Gao

**Affiliations:** ^1^Department of Rehabilitation, Third Affiliated Hospital of Zhejiang Chinese Medical University, Hangzhou, China; ^2^The Third Clinical Medical College of Zhejiang Chinese Medical University, Hangzhou, China; ^3^Department of Acupuncture, Third Affiliated Hospital of Zhejiang Chinese Medical University, Hangzhou, China

**Keywords:** post-stroke depression, integrative medicine, acupuncture, traditional Chinese medicine, repeated transcranial magnetic stimulation

## Abstract

**Background:**

Post-stroke depression (PSD) is one of the most common neuropsychiatric diseases in patients with stroke, and it can increase the disability rate, mortality, and recurrence rate of stroke. Currently, many clinical studies have indicated that traditional Chinese medicine (TCM), such as acupuncture and herbs, Western medicine, rehabilitation, repeated transcranial magnetic stimulation, and other treatment methods, are effective in treating PSD. However, no study has formulated a comprehensive treatment plan that integrates TCM, Western medicine, and rehabilitation for PSD. Thus, this trial aims to assess the efficacy and safety of integrative medicine for treating PSD.

**Methods:**

This multicenter, prospective, randomized, controlled study aims to form a set of effective clinical treatment schemes that integrate TCM, Western medicine, and rehabilitation for PSD. A total of 202 participants recruited from four centers will be randomized into either the integrative medicine or standard care group. Standard care—basic treatment, general nursing care, and exercise therapy—will be provided to all participants. The integrative medicine group will also receive acupuncture, Chinese herbs, and repeated transcranial magnetic stimulation (rTMS). Participants will receive acupuncture and rTMS treatments five times per week for 4 weeks and will be administered Chinese herbs, basic treatment, general nursing care, and exercise therapy for 4 weeks. The primary outcomes include the Hamilton Depression Scale (HAMD), Self-Rating Depression Scale (SDS), and Activity of Daily Living Scale (ADL). And the secondary outcomes include the Montreal Cognitive Assessment Scale, the Fugl-Meyer Assessment (FMA) Scale, and the Pittsburgh Sleep Quality Index (PSQI). All outcome measures will be evaluated at baseline, week 4 (the end of the treatment courses), and week 8 (the end of follow-up). Safety assessments will be performed throughout the study.

**Discussion:**

This study is expected to verify the efficacy and safety of integrative medicine for treating PSD, providing an evidence-based clinical reference for the future development of a standardized scheme.

**Clinical trial registration:**

ClinicalTrials.gov, identifier: NCT05187975

## Introduction

Stroke has become the world's second most common cause of death (Mozaffarian et al., 2016). According to comprehensive global, regional, and national stroke lifetime risk data, the lifetime risk of stroke for people aged 25 or more in 2016 was 24.9% (Feigin et al., 2018). The overall risk of lifelong stroke in the Chinese population is as high as 39.3% (Wang et al., 2019). Moreover, post-stroke depression (PSD) is the most frequent neuropsychiatric sequela of stroke. Many patients suffer from depression after a stroke. Statistically, the incidence varies from one study to another, with the estimated frequency of PSD being 31% (Hackett and Pickles, 2014).

Antidepressants are currently the main treatment for depression. However, the side effects of Western drugs on the human nervous system, digestive system, liver, and biliary system are inevitable. Nonpharmacological interventions include psychotherapy, exercise therapy, acupuncture, and noninvasive brain stimulation, among others (Lanctôt et al., 2020). Acupuncture has been demonstrated to be beneficial for treating PSD. For example, a previous meta-analysis found that in conjunction with conventional acupuncture, scalp acupuncture can improve depression and promote the activities of daily living (ADLs) in PSD patients, thereby indicating that acupuncture may be a promising therapy for PSD (Hang et al., 2021). Traditional Chinese medicine (TCM), such as Chinese herbs, has certain curative effects on the depressive state, neurological deficits, and daily living ability of PSD patients. Clinically, liver-Qi stagnation is one of the most common syndrome patterns of PSD according to the TCM syndrome patterns. The Chinese herb “Chaihu Shugan Powder” is one of the representative formulas for treating liver-Qi stagnation syndrome. Previous studies have reported that Chaihu Shugan powder could relieve the depressive state in patients with PSD (Tao, 2015; Yuan, 2018). In addition, repeated transcranial magnetic stimulation (rTMS) is recognized by the U.S. Food and Drug Administration (FDA) because it is safe and effective with minimal side effects (Perera et al., 2016), and depression is a recommended indication of rTMS (Level A evidence) (Lefaucheur et al., 2020). In addition, rTMS effectively improves the daily living ability and cognitive function of patients with PSD (Shen et al., 2017). Thus, it is anticipated that the above TCM therapies and rTMS can broaden the treatment approach for PSD. Meanwhile, they can bring adjunctive therapeutic effects to conventional treatments with enhanced safety profiles. Thus, they may be worthy of clinical applications and vigorous development.

In reference to the previous literature, most clinical studies have reported that when a specified TCM or rehabilitation therapy is combined with drug therapy or other active treatments, such integrated approaches can generally lead to higher efficiency and fewer adverse effects (Wen et al., 2016; Qiao et al., 2020). The advantage of integrative medicine is that it makes full use of all appropriate therapeutic approaches (including conventional and complementary therapies), health care professionals, and disciplines to achieve optimal efficacy. Nonetheless, despite its potential superiority, the concept of “integrative medicine” is often underestimated, and to date, clinicians have not taken full advantage of integrative medicine in clinical settings (Liu et al., 2019).

Taken together, we designed this multicenter, prospective, randomized, controlled study to assess the efficacy and safety of integrative medicine on PSD, which integrates TCM, Western medicine, and rehabilitation treatment. To the best of our knowledge, this is the first study to involve a combination of multiple well-established rehabilitation therapies for PSD under the increasingly popular concept of integrative medicine. It is expected that our study will contribute to forming a standardized treatment scheme of integrative medicine for PSD in the future.

## Methods

### Study design

**Figure 1:**
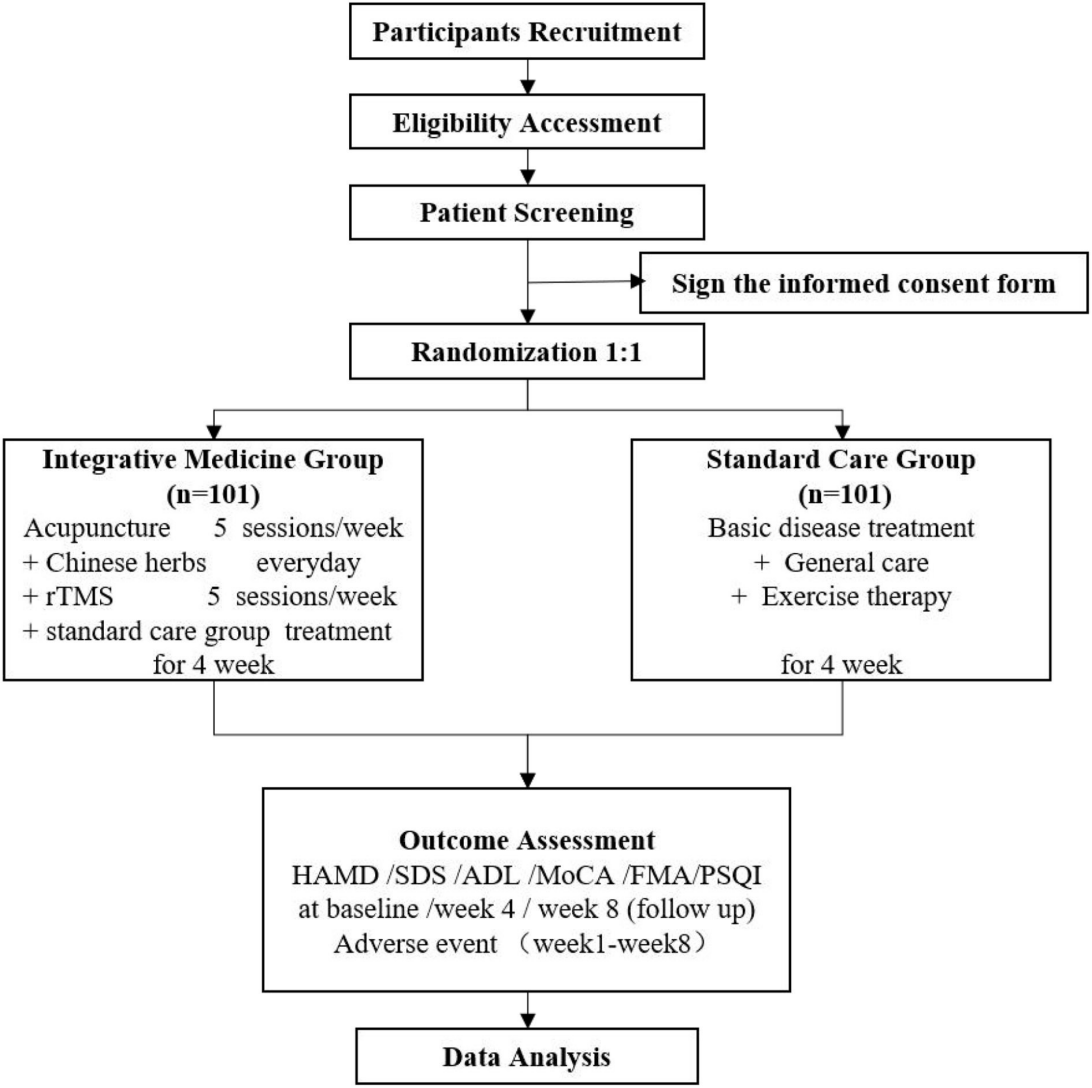
Flowchart of the trial. rTMS, Repeated Transcranial Magnetic Stimulation; HAMD, Hamilton Depression Scale; SDS Self-Rating Depression Scale; ADL, Activities of Daily Living; MoCA, Montreal Cognitive Assessment scale; FMA, the Fugl-Meyer Assessment of Motor Function Scale.

This study is designed as a multicenter, prospective, parallel-group, randomized, controlled trial (RCT). All eligible participants will be randomly assigned to either the integrative medicine or standard care group. The study duration will be 12 weeks, which consists of a 4-week treatment period and an 8-week follow-up period. The research flow chart is displayed in Figure 1, and the trial schedule of enrollment, treatments, and assessments is displayed in Table 1. The reporting of this protocol is strictly based on the Standard Protocol Items: Recommendations for Interventional Trials (SPIRIT) (Chan et al., 2013).

**Table 1:** Schedule of enrollment, treatments, and assessments.

**Time (weeks)**	**W-1**	**W 0**	**W 1**	**W2**	**W3**	**W4**	**W 8**
**Period assessment**	**Screening**	**Baseline**	**Intervention**				**Follow-up**
**Patients**
Screening	×						
Informed consent	×						
Demographic data		×					
Disease history		×					
Randomization		×					
**Intervention**
Integrated rehabilitation group (*n* = 101)			Acupuncture TCM rTMS add the same treatment of the standard care group				
Standard care group (*n* = 101)			Basic disease treatment General care Exercise therapy				
**Primary outcomes**
HAMD		×	×				×
SDS		×	×				
ADL		×	×				×
**Secondary outcomes**
MoCA		×	×				
PSQI		×	×				×
FMA		×	×				
Adverse events			×	×	×	×	×
Causes of dropout			×	×	×	×	×

### Ethical approval and study registration

This study was approved by the Medical Ethics Committee of the Third Affiliated Hospital of Zhejiang Chinese Medical University and registered with the ClinicalTrials.gov database (NCT Number: NCT05187975).

### Patients and enrollment

#### Diagnostic criteria

Participants should meet the diagnostic criteria of cerebral infarction (Rao, 2010) or cerebral hemorrhage (Rao, 2010) in combination with depressive disorders (Psychiatry CSo, 2001). Meanwhile, the TCM syndrome pattern should meet the diagnostic standard (Yu and Fang, 2018) of liver-Qi stagnation syndrome according to the following aspects:

(1) qualified tongue and pulse conditions of TCM: the patient has a pale red tongue, thin and greasy moss, and a wiry pulse;
(2) primary symptoms and signs include mental depression, emotional disturbance, frequent sighing, thoracic oppression, fullness, or distending pain in the hypochondrium, migratory pain, and epigastric distention; and
(3) secondary symptoms and signs include no appetite, irregular bowel movements, and irregular menstrual periods (for females);

With the qualified tongue and pulse conditions, if a patient has three primary symptoms/signs or two secondary symptoms/signs combined with two primary symptoms/signs, they may be diagnosed with liver-Qi stagnation syndrome.

#### Inclusion criteria

(1) participants who meet the aforementioned diagnostic criteria;
(2) consciousness, stable vital signs, and ability to understand and follow the instructions. Barthel Index (BI) > 20 (Mahoney and Barthel, 1965), FMA (0–95), Mini-mental State Examination (MMSE) meet the following criteria: illiteracy > 17, primary school level > 20, secondary school level (including technical secondary school) > 22, and college-level (including junior college) > 23 points;
(3) 25 ≤ age ≤ 85, regardless of gender;
(4) first episode of stroke;
(5) depression level as mild or moderate (HAMD scores ≥ 7 and ≤ 24);
(6) the course of the disease is limited to 2 weeks to 36 months after the stroke; and
(7) participants can understand the study protocol and provide written informed consent.

#### Exclusion criteria

(1) patients with acute brain trauma, brain infection, effusion, or tumor occupation;
(2) there are intracranial metals and other foreign bodies (such as orthopedic materials, arterial clips, etc.), cardiac pacemakers, deep brain stimulators, and other electronic devices;
(3) patients with previous seizures, including primary and secondary seizures;
(4) patients with severe complications of the cardiovascular system, liver, kidney, or a psychiatric history;
(5) there is a significant cognitive impairment [MMSE: illiteracy ≤ 17, primary school level ≤ 20, secondary school level (including technical secondary school) ≤ 22, and college-level (including junior college) ≤ 23 points] or hearing impairment, aphasia;
(6) patients have coma, terminal illness, or acute exacerbation of chronic disease;
(7) patients who have taken psychotropic drugs or have already been treated for depression for nearly a month; and
(8) people with unstable vital signs or patients with other mental disorders.

#### Discontinuation criteria

(1) serious adverse reactions have occurred during the study;
(2) subjects develop serious complications or other serious diseases during the study, and emergency measures are needed; and
(3) the subject is unable to continue the study for other reasons.

#### Patient recruitment

In this RCT, we will recruit hospitalized patients from acupuncture departments, rehabilitation departments, and neurology departments in four trial centers, which are the Third Affiliated Hospital of Zhejiang Chinese Medical University, Hangzhou Hospital of Traditional Chinese Medicine, the Second Hospital of Jinhua, and Zhejiang General Hospital of Armed Police.

### Randomization and allocation

The randomization method for this study is a central randomization system. An external statistician will use randomizer software (SPSS software version 25.0) to generate a table of random numbers. Applications for random numbers can be made *via* text messages or phone calls. The applicant will call the randomization center and then enter the subject information in the voice form or send the subject information to the randomization center by SMS. After the central randomization system receives the application information, the patient's randomization number and the group will be returned, and the applicant will receive the randomization assignment email. Grouping information will be hidden using sequentially numbered, opaque, and sealed envelopes to achieve allocation concealment.

### Blinding

Although unable to blind the intervention operators and participants due to the unique nature of the complex interventions in our trial, the operators, outcome assessors, and statistical analysts will be separated and performed by respective specially-assigned persons, thereby ensuring blinding principles during the process of our clinical trial. The outcome measurement and statistical analyses will be carried out by specially designated researchers who do not know the grouping to avoid possible bias as much as possible.

### Sample size calculation

The data statistician will carefully compute the appropriate sample size for each group based on the difference in postintervention HAMD scores between the two groups. According to our unpublished pilot study, the HAMD score (mean ± standard deviation) in the standard care group was 16.5 ± 4.51, and the HAMD score in the integrative medicine group was 14.5 ± 3.40. The mean HAMD score in the integrative medicine group was 2 points lower than that in the standard care group. Based on a 5% false-positive error rate (α = 0.05, two-sided, the normal distribution quantile table shows that *Z*_1−α/2_ = 1.96) and 90% power (β = 0.1, the normal distribution quantile table shows that *Z*_1−β_ = 1.28), the sample size is estimated as follows:


$$\begin{array}{l} \begin{array}{l} n=\frac{{\left(Z_{1-\alpha /2}+Z_{1-\beta }\right)}^{2}\times (\sigma_{1}^{2}+\sigma_{2}^{2})}{\delta^{2}}\\ \;=\frac{{\left(1.96+1.28\right)}^{2}\times (3.40^{2}+4.51^{2})}{2^{2}}\approx 83.72 \end{array} \end{array}$$

Each group needs to include 84 participants. Assuming an ~20% dropout rate, a total of 202 participants will be enrolled in this trial.

### Interventions

All qualified participants will be randomly assigned to one of two groups: the integrative medicine group or the standard care group. Standard care—comprising basic treatment, general nursing care—and exercise therapy will be provided to both groups. The integrative medicine group will also receive acupuncture, Chinese herbs, and rTMS. Participants will receive acupuncture and rTMS treatments five times per week for 4 weeks and receive Chinese herbs, basic treatment, general nursing care, and exercise therapy for 4 weeks. All treatments will be carried out by expert acupuncturists and rehabilitation therapists, who will follow standard operating protocols to ensure that each patient receives the same treatments.

The intervention is described following the Template for Intervention Description and Replication (TIDieR) checklist:

#### Integrative medicine group

In addition to standard care that is the same as the control group, the integrative medicine group will receive acupuncture, Chinese herbs, and rTMS.

(1) Acupuncture:

The acupuncture method of the trial will comprise a combination of scalp acupuncture (SAC NTCoAaMo, 2021) and body acupuncture.

Scalp acupuncture includes the middle line of the forehead (MS1), the front line by the forehead (MS2), and the middle line of the vertex (MS5). These acupoints are needled for 1 inch with the direction of the scalp tilted 15–30 degrees. The scalp needles are kept for 1 h, with two times the intermittent manipulation of the needle, 10 s, 200 r/min. The treatment frequency is one time per day and five times per week across 4 weeks.

Body acupuncture points include Yintang (GV29), Taichong (LR3), Shenmen (HT7), Neiguan (PC6), Danzhong (CV17), Qimen (LR14), and Taixi (KI3). After local disinfection, the Yintang (GV29) acupoint is needled for 0.3–0.5 inches with the horizontal insertion of the needle direction. The bilateral Taichong (LR3) acupoints are needled for 0.5–0.8 inches in the straight direction. The Danzhong (CV17) and bilateral Shenmen (HT7) acupoints are needled for 0.3–0.5 inches in the straight direction. The bilateral Neiguan (PC6) and Taixi (KI3) acupoints are needled for 0.5–1 inch in the straight direction. The bilateral Qimen (LR14) acupoint is needled for 0.5–0.8 inches with the horizontal insertion of needle direction. The body needles are kept for 30 min; the treatment frequency is five times per week, and the treatment duration is 4 weeks.

(2) Chinese herbs

The Chinese herbs called “Chaihushugan powder” include the following prescriptions (Yu and Fang, 2018): Chaihu 12 g, Chenpi 12 g, Chuanxiong 9 g, vinegar Xiangfu 9 g, Zhike 9 g, Shaoyao 9 g, and roasted Gancao 3 g.

The dosage is to take the granules with 100 ml of warm water two times a day (after breakfast and dinner) for 4 weeks.

(3) rTMS

rTMS equipment: CCY-I-type magnetic field stimulator

rTMS protocol: The stimulus is applied over the left dorsolateral prefrontal cortex (DLPFC) and the left temporal lobe. The stimulation parameters are as follows: 100% magnetic field strength relative to the patient's observed resting motor threshold, at 10 pulses per s for 5 s, with an intertrain interval of 50 s. One treatment session lasts 30 min (30 trains) and comprises 1,500 pulses. The frequency and treatment course of rTMS is five times per week for 4 weeks.

#### Standard care group

(1) Basic pharmacological treatment

The specific methods of the basic pharmacological treatment are formulated concerning the *Guidelines for prevention and treatment of cerebrovascular diseases in China (2010 Edition)*. When appropriate, corresponding drugs that have the effect of lipid regulation, blood sugar control, antihypertension, and anticoagulation will be used depending on the patient's primary diseases and complications. The antidepressant drug is paroxetine tablets (Beijing Fuyuan Pharmaceutical Co., Ltd., Guoyao Zhunzi: h20133084) 20 mg, taken orally after meals every morning.

(2) General nursing care

The nursing staff will assess the general condition, psychological status, and family status of the patients. They will also strengthen guardianship, communicate with the patients, reduce insecurity of the patients, and prevent unsafe incidents such as suicide. Nursing care will be provided to keep the patients' sleep, diet, and environment comfortable.

(3) Exercise therapy

A professional therapist will assess the patient's Brunnstrom stage. In accordance with the impairment of the patient's motor functions (e.g., muscle tone, muscle strength, balance, movement coordination, and proprioception), an individualized scheme of exercise therapy will be developed. The scheme of exercise therapy includes proper limb positioning, joint range-of-motion training, muscle strength training, turnover, transfer training, sitting, standing balance training, gait training, and so on. One session will last for 30–45 min. The treatment frequency and treatment course of exercise therapy will be once a day for 4 weeks (Yu and Park, 2013; Bang and Son, 2016).

Standard care (including basic pharmacological treatment, general nursing care, and exercise therapy) is a fundamental treatment for stroke and PSD. It is worth noting that after receiving 4 weeks of the aforementioned standard care in both the integrative medicine and standard care groups, specialist physicians will determine whether such standard care approaches should be continued based on the individual recovery conditions.

### Outcome measures

#### Primary outcomes

The primary outcome will be measured at baseline, week 4 after treatment, and week 8 during follow-up.

##### Hamilton depression scale

The HAMD, originally published by Max Hamilton (Hamilton, 1960), is a standard efficacy outcome in antidepressant clinical trials (Chagas et al., 2010). The participants will be rated on 17 dimensions on a 2- or 4-point scale. The higher the score, the more serious the condition of depression. A score of 0–7 is considered to have depressive symptoms, a score of 8–17 is considered to be possible depression, a score of 18–24 is considered to be clear depression, and a score of 25–52 is considered to be severe depression. It takes ~20 m to complete the assessment.

##### Self-rating depression scale

The self-rating depression scale was developed by W.K. Zung in 1965. It can reflect the depressive state and its variability well (Zung, 1965). The scale contains 20 items scored on a 4-point scale. The rough scores were obtained by summing each of the 20 new items, multiplying the rough scores by 1.25, and then taking the integer part as the standard score. A standard score of 50–59 is considered to be a mild depressive mood, a standard score of 60–69 is considered to be moderate depression, and a standard score of more than 70 is considered to be severe depression. Patients respond according to their situation in the past week.

##### Activity of daily living scale

The ADL was developed in 1969 by Lawton and Brody in the U.S. (Lawton and Brody, 1969). The scale has two parts: the Physical Self-Maintenance Scale and the Instrumental Activities of Daily Living Scale. It is principally used to assess the subject's ability to perform daily living activities. The range of scores is from 20 to 80, with higher scores indicating more functional impairment (Liu et al., 2018). A score of more than 23 is considered a cognitive impairment.

#### Secondary outcomes

The secondary outcome will be measured at baseline, week 4 after treatment, and week 8 during follow-up.

##### Montreal cognitive assessment scale

The MoCA was developed based on clinical experience and concerning the Mini-Mental State Examination. It is a common way to assess a tester's awareness and is more sensitive to detecting mild cognitive impairment. Not only that, but the MoCA and its subtests are reliable and valid for assessing global and specific cognitive performance in patients with depression, and it could be a tool for screening neurocognitive deficits in depressed patients (Srisurapanont et al., 2017). The scale covers eight cognitive domains, including attention and concentration, executive function, memory, language skills, visual structure, abstract thinking, numeracy, and orientation, among other 11 items. The total score is 30 points. Add 1 point if the number of years of education is ≤12 years, and a score of ≥26 is considered normal.

##### The Fugl-Meyer assessment scale

The FMA scale is one of the most extensively used and recognized methods for assessing motor function (Rech et al., 2020), with a score of 100 for motor function (66 for upper extremity motor function and 34 for lower extremity motor function). The higher the score, the better the motor function.

##### Pittsburgh sleep quality index

The PSQI was developed in 1989 by Buysse et al. (1989), a psychiatrist at the University of Pittsburgh, to assess the quality of sleep of subjects in the last month. This scale can be used not only to assess sleep behavior and habits in the general population but also has a high level of reliability and validity (Mollayeva et al., 2016). It can be used for the general evaluation of sleep quality in clinical patients. A score of 0–5 is considered to be good sleep quality, a score of 6–10 is considered to be OK sleep quality, a score of 11–15 is considered to be average sleep quality, and a score of 16–21 is considered to be very poor sleep quality.

### Safety assessment

To avoid AEs, such as local bleeding or discomfort at the acupuncture points, local redness or bruising, itching, and dizziness, the participants' safety will be monitored throughout the treatment. The timing of onset, intensity, progression, and management of the AEs will be meticulously noted at each visit. If a major adverse event occurs, the primary investigator and the institutional review board will be notified as soon as possible, and immediate action will be taken.

### Data collection and management

In this study, case report form (CRF) tables will be used to collect data. The assessment of the HAMD and SDS scales will be completed by psychiatrists, and trained physicians will complete the other scales through conversations and physical examination. Among them, the scale assessment for the follow-up period will be performed by telephone follow-up. The data manager is specifically trained in transcribing the data from CRFs to the database used for the statistical analyses.

### Statistical analysis

Statistical analyses will be carried out by statisticians unaware of the groupings. The statistical analysis will be performed using SPSS software version 25.0. A two-sided 5% significance level will be adopted, and the corresponding 95% CI will be calculated to the greatest extent possible. A *p*-value of <0.05 will be set as the significance level.

Demographic characteristics and baseline measurements of the variables in each group will be summarized. Measurement data that conform to a normal distribution will be displayed using the mean ± SD, while the median, minimum, and maximum will describe measurement data that do not conform to a normal distribution. To assess the treatment effectiveness, outcome measurement data at baseline, after the intervention, and follow-up (i.e., 4 and 8 weeks), will be analyzed. For the qualitative data, the difference between and within groups will be tested by the chi-square or Fisher's exact test. For quantitative data, either parametric tests (Student's *t*-test) or non-parametric tests (Mann–Whitney *U*-test, Wilcoxon signed-rank test) will be used, depending on the normality of the distribution. In addition, the intention-to-treat analysis will be carried out to minimize potential attrition bias.

### Quality control

To minimize variations during the intervention process and outcome assessment process, quality control measures will be taken. Prior to the trial, all manipulators who deliver the interventions, as well as the outcome assessors, will attend intensive training about the routine standard operating procedure (SOP) and quality assurance programs, thereby improving the homogeneity. Moreover, during the trial, experts will visit each center to inspect the quality of the outcome assessment and the standardization of intervention schemes for quality assurance.

In addition, we will try to reduce the actual dropout rate by the following strategies. First, based on training, the research team will try their best to improve the quality of treatment and physician–patient communication. Second, when participants miss a visit, the research team will call them to remind them about their visit promptly. Third, the participants will receive acupuncture, Chinese herbs, and rTMS free of charge.

## Discussion

Studies have indicated that TCM (e.g., acupuncture and Chinese herbs), rehabilitation, and rTMS effectively improve the symptoms of PSD. However, it is notable that most previously published clinical studies have reported higher efficiency and fewer adverse effects when a TCM or rehabilitation therapy is added to a Western medicine control group or a control group with other therapies; however, most previous trials only added one or two TCM therapies. To date, very few clinical trials have examined the treatment of PSD that focus on the concept of integrative medicine in real-world clinical settings. Notably, in most Asian countries, especially China, aiming to optimize the therapeutic effect through closer collaborations between TCM and Western medicine, clinicians often adopt an integrative medicine approach to treat a wide range of diseases in hospitals, rather than just a sole TCM or Western medicine approach. Therefore, we designed an integrative medicine treatment protocol incorporating a total of three therapies—namely acupuncture, Chinese herbs—and rTMS for PSD. Our integrated medicine protocol incorporating the aforementioned therapies is commonly used and has promising application value for PSD in real-world clinical settings, especially in Asian hospitals, so we intend to investigate its therapeutic effect. Taken together, our study has a certain innovative quality to it.

According to TCM theory, the liver-qi stagnation pattern is the leading TCM pathogenesis of PSD. Soothing the liver and regulating qi is the treatment principle. Therefore, in terms of acupoint selection, the scalp acupoints are MS1, MS2, and MS5, which can treat mental diseases according to the expert consensus entitled *Standardized manipulations of acupuncture and moxibustion-Part 2: Scalp acupuncture* (SAC NTCoAaMo, 2021). The MS1, MS2, and MS5 are placed on the top of the head, the stimulation of which can exert local and nearby therapeutic effects. Based on *Clinical Acupuncture*, the body acupoints chosen for this study are the most commonly and normatively used by clinical practitioners. They are effective in tranquilizing the mind and relieving liver stagnation. In terms of herbal medicine, “Chai Hu Shu Gan San” has some benefits in treating depression, particularly PSD (Sun et al., 2018). In this formula, ChaiHu is the sovereign drug, and XiangFu and ChuanXiong are the minister drugs that play a role in promoting Qi and relieving depression together with ChaiHu. In addition, ChaiHu has neuroprotective effects (Li et al., 2016), XiangFu has also been found to have anti-inflammatory activity in both the peripheral and central nervous systems (Azimi et al., 2016; Farahani and Hashemi, 2016; Johari et al., 2016), and ChuanXiong is a classic herb with anti-inflammatory effects in treating cardiovascular and cerebrovascular diseases (Cao et al., 2016; Liu et al., 2016). Its conventional activity tends to be neuroprotective from a translational medicine perspective (Lin et al., 2009; Lu et al., 2014). Therefore, the prescription of Chai Hu Shu Gan San is effective in improving depression-associated neurological symptoms. rTMS is applied over the prefrontal cortex. It induces a magnetic field that results in the depolarization of underlying neurons (Wassermann and Zimmermann, 2012) and the modulation of the neural circuitry involved in emotion regulation and depressive symptoms (Barker et al., 1985, 1986; George et al., 1995; Liston et al., 2014; Salomons et al., 2014). The safety and efficacy of rTMS antidepressant therapy have been supported by multiple randomized controlled trials and published literature. These consensus recommendations, developed by the NNDC rTMS Task Group and the APA CoR Task Force on Novel Biomarkers and Treatments, provide comprehensive information for the safe and effective clinical application of rTMS in treating MDD (McClintock et al., 2018). Moreover, a previous study reported that patients with treatment-resistant depression had positive experiences and attitudes toward rTMS [Ontario Health (Quality), 2021].

However, some limitations of this study should be addressed. First, the outcome indicators used in this study are mainly patient-reported scales, which may affect the results due to the subjectivity of the patients' responses. Therefore, multiple scales are adopted simultaneously to assess the patients' depression on a multidimensional level. Second, due to limited funds, the duration of the follow-up is not long enough. Third, although the design of study groups in our study focuses more on the concept of “integrative medicine” in real-world clinical settings and the treatment protocol in these two groups is commonly used for PSD in Asian hospitals, the lack of setting a solo treatment group (e.g., acupuncture group, rTMS group) may weaken the expected findings to a certain extent.

In summary, based on this multicenter RCT with a large sample size and sound methodology, the efficacy of integrative medicine for PSD will be evaluated. Given that the integrative medicine scheme for PSD in our study integrates both TCM and Western medicine and incorporates conventional and complementary therapies, it is expected that our study will contribute to forming a standardized treatment scheme for integrative medicine for PSD in real-world clinical settings in the future.

## Ethics statement

The studies involving human participants were reviewed and approved by the Medical Ethics Committee of the Third Affiliated Hospital of Zhejiang Chinese Medical University. The patients/participants provided their written informed consent to participate in this study.

## Author contributions

JC and HG conceived the study, participated in its design and coordination, and helped to draft the manuscript. KS and LF participated in the study design and made substantial contributions to the manuscript drafting. HH, TL, and YZ participated in the study design and revised the draft critically. All authors read and approved the final manuscript.

## Funding

The trial was financially funded by the 2021 Special Project for Modernization of Chinese Medicine in Zhejiang Province (Grant No. 2021ZX010), Zhejiang Provincial Famous Traditional Chinese Medicine Experts Inheritance Studio Construction Project (Grant No. GZS2021027), and Zhejiang Province Public Welfare Technology Project (Grant No. LGF21H270007).

## Conflict of interest

The authors declare that the research was conducted in the absence of any commercial or financial relationships that could be construed as a potential conflict of interest.

## Publisher's note

All claims expressed in this article are solely those of the authors and do not necessarily represent those of their affiliated organizations, or those of the publisher, the editors and the reviewers. Any product that may be evaluated in this article, or claim that may be made by its manufacturer, is not guaranteed or endorsed by the publisher.
